# A 2-nt RNA enhancer on exon 11 promotes exon 11 inclusion of the Ron proto-oncogene

**DOI:** 10.3892/or.2013.2835

**Published:** 2013-11-05

**Authors:** HEEGYUM MOON, SUNGHEE CHO, TIING JEN LOH, JIANHUA ZHOU, CLAUDIA GHIGNA, GIUSEPPE BIAMONTI, MICHAEL R. GREEN, XUEXIU ZHENG, HAIHONG SHEN

**Affiliations:** 1 School of Life Sciences, Gwangju Institute of Science and Technology, Gwangju 500-712, Republic of Korea; 2 Jiangsu Key Laboratory of Neuroregeneration, Nantong University, Nantong 226001, P.R. China; 3 Institute of Molecular Genetics, National Research Council, I-27100 Pavia, Italy; 4 Howard Hughes Medical Institute and Programs in Gene Function and Expression and Molecular Medicine, University of Massachusetts Medical School, Worcester, MA 01605, USA

**Keywords:** Ron, proto-oncogene, cancer, pre-mRNA splicing, exon 11 inclusion, *cis*-elements

## Abstract

Ron is a human receptor for the macrophage-stimulating protein (MSP). Exon 11 skipping of Ron pre-mRNA produces the RonΔ165 protein that has a deletion of a 49 amino acid region in the β-chain extracellular domain. RonΔ165 is constitutively active even in the absence of its ligand. Through stepwise deletion analysis, we identified a 2-nt RNA enhancer, which is located 74 nt upstream from the 5′ splice site of exon 11, for exon 11 inclusion. Through double-base and single-base substitution analysis of the 2-nt RNA, we demonstrated that the GA, CC, UG and AC dinucleotides on exon 11, in addition to the wild-type AG sequence, function as enhancers for exon 11 inclusion of the Ron pre-mRNA.

## Introduction

The RON receptor tyrosine kinase along with c-Sea, c-Met and Stk are members of the MET proto-oncogene family (1). The RON gene consists of 20 exons (2). RON protein is a 180-kDa heterodimeric protein composed of a 40-kDa α chain and a 150-kDa β chain linked by disulfide bonds (3). While the α chain contains the extracellular domain for ligand binding, the β chain includes the intracellular part that contains a kinase domain and a transmembrane domain (4). These two chains are derived from the 180-kDa precursor protein by a proteolytic cleavage (5). The macrophage stimulating protein (MSP) was first identified as a ligand for Ron protein (6). MSP binds to the RON receptor to upregulate RON kinase activity, which leads to autophosphorylation on the tyrosine residues in the kinase domain and the C-terminal docking site (7–9). Activation of the RON receptor by MSP stimulates a large number of downstream intracellular pathways (10). Tumor formation and progression occurs when the accumulation and activation of receptor tyrosine kinases are abnormal (11). RON overexpression and activation induce tumor progression and invasive growth of certain types of epithelial tumor cells (12,13). Alternative splicing of Ron pre-mRNA produces various protein isoforms (14). RONΔ165 protein, identified in gastric cancer cell line KATOIII, is generated by skipping of exon 11 (15). RONΔ165 does not undergo proteolytic processing and is retained intracellularly. Furthermore, uneven numbers of cysteine residues in RONΔ165 produces the RON oligomer. Therefore, RONΔ165 is constitutively activated without the binding of the MSP ligand. Abnormal accumulation of this isoform was found in some types of breast and colon cancer cell lines (16). Furthermore, overexpression of this splice variant can induce invasive growth and metastasis (15). Apart for the fact that ASF/SF2 induces skipping of exon 11 to control cell motility (16), the splicing mechanism of Ron exon 11 is not yet well understood.

Pre-mRNA splicing is a process in which introns are removed and then exons are ligated (17–19). The RNA sequences required for splicing are called splicing signals that include the 5′ splice site, 3′ splice site, branch point and polypyrimidine tracts (PPT) (20). In the alternative splicing procedure, different splicing signals are selected to produce multiple mRNA isoforms from a single gene through the numerous combinations of multiple exons (21). Alternative splicing is one of the critical mechanisms for gene regulation that generates proteomic diversity (22,23). Abnormal regulation of alternative splicing causes a variety of human diseases including cancer (24). Alternative splicing is finely regulated by several *cis*-acting elements and *trans*-acting elements (25,26). *cis*-acting elements are RNA sequences on pre-mRNA that function as either enhancers or inhibitors to regulate exon inclusion or skipping. Some *cis*-elements provide binding sites for SR proteins and hnRNP proteins to regulate splicing. Juxtaposed enhancers and inhibitors functionally antagonize each other (27,28). Exon 11 inclusion of Ron pre-mRNA is regulated by a juxtaposed enhancer and inhibitor on exon 12 (16). In the present study, we showed that exon 11 of Ron pre-mRNA also contains various *cis*-regulating elements for exon 11 inclusion. Specifically, a 2-nt RNA, located at 74 nt upstream from the 5′ splice site of exon 11, functions as an enhancer for exon 11 inclusion. Through double base and single base substitution analysis on the 2-nt RNA, we demonstrated that the GA, CC, UG and AC dinucleotides on exon 11, in addition to the wild-type AG sequence, function as enhancers for exon 11 inclusion of Ron pre-mRNA.

## Materials and methods

### Construction of plasmids

The wild-type RON exon 10–12 sequences were amplified from human genomic DNA using RON10-*Hin*dIII-for and RON12-*Xho*I-rev primers (Table I) and cloned into *Hin*dIII and *Xho*I restriction enzyme sites of the pCDNA3.1 (+) vector. Every deletion and mutation construct was produced with overlapping PCR. All primers used for minigene constructs are listed in Table I.

### Cell culture and transfection

MDA-MB-231 cells were grown in RPMI-1640 medium supplemented with 10% fetal bovine serum (FBS) at 37°C in a humidified 5% CO_2_ atmosphere. Ron minigene transfection into MDA-MB-231 cells was carried out with polyethyleneimide (PEI) according to the manufacturer’s protocol.

### RT-PCR

Total RNA was extracted from the MDA-MB-231 transfected cells using RiboEx reagent (GeneAll, Korea) following the manufacturer’s protocol. Total RNA (1 μg) was reverse transcribed using oligo dT_18_ using ImProm-II™ reverse transcriptase (Promega, Madison, WI, USA) following the manufacturer’s protocol. cDNA (1 μl) was amplified by PCR using G-Taq polymerase (Cosmo Genetech, Seoul, Korea). RON minigenes were as following: RON10-forward (5′-CCTGGCTTTCGCTTCCTACC-3′) and pCDNA-reverse (5′-CTAGAAGGCACAGTCGAGGCT-3′). GAPDH primer sequences were as following: GADPH-forward (5′-ACCACAG TCCATGCCATCA-3′) and GAPDH-reverse (5′-TCCACC ACCCTGTTGCTGTA-3′).

## Results

### Exon 11 contains various regulatory elements for exon 11 inclusion of Ron pre-mRNA

**Figure 1:**
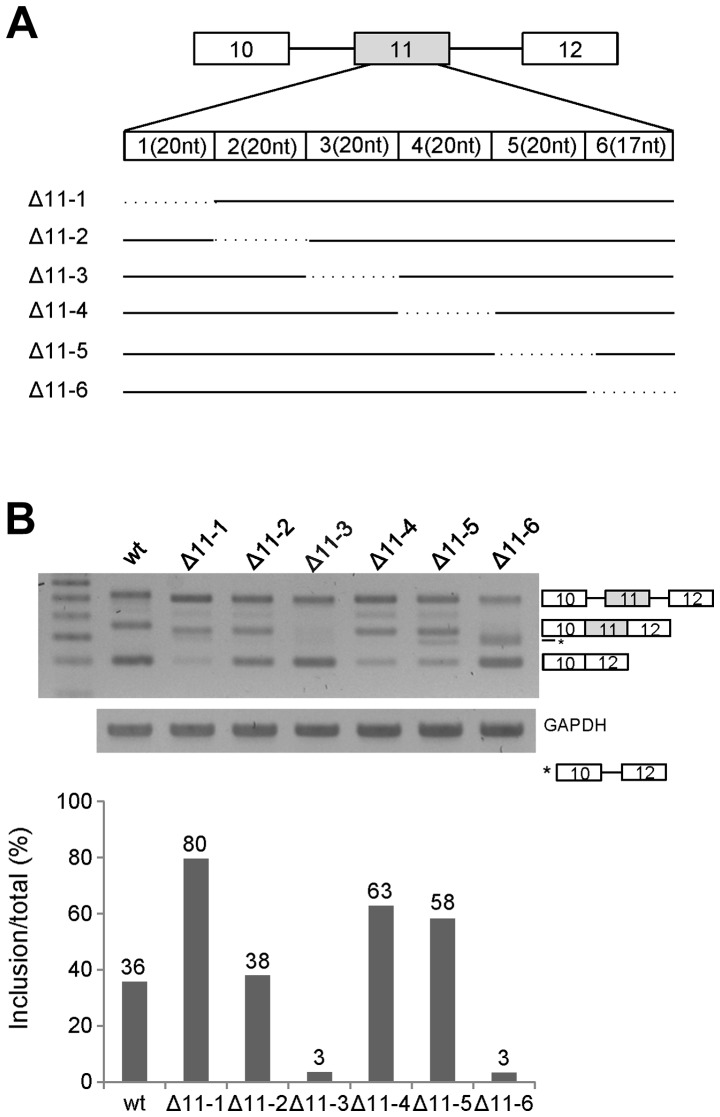
Exon 11 contains various regulatory elements for exon 11 inclusion of Ron pre-mRNA. (A) A series of deletion mutants (Δ11-1, Δ11-2, Δ11-3, Δ11-4, Δ11-5 and Δ11-6) is shown. The lengths of each section are indicated. Dotted lines indicate the deleted sections for each mutant. (B) RT-PCR analysis of wild-type (wt) minigene and deletion mutants. The partially spliced product is marked with a star. GAPDH was used as a control. Quantitation of the results is shown as a ratio of exon 11 inclusion to total RNA.

In order to identify the enhancer on exon 11 for exon 11 inclusion of Ron pre-mRNA, we performed mutagenesis analysis on exon 11. In the first step, we divided exon 11 into six parts (11-1, 11-2, 11-3, 11-4, 11-5 and 11-6) with the first five parts containing 20-nt RNA in each and the last part containing 17-nt RNA. The first and last parts are located 15 nt apart from the 3′ and 5′ splicing sites of exon 11 (Fig. 1A). We produced deletion mutants for each part of RNA named as Δ11-1, Δ11-2, Δ11-3, Δ11-4, Δ11-5 and Δ11-6. We extracted RNA from the mutant minigene-transfected cells, and then performed RT-PCR analysis for Ron exon 11 splicing on each mutant. As shown in Fig. 1B, exon 11 splicing of Δ11-2 had the similar level of exon 11 inclusion as that of the wild-type. However, exon 11 inclusion was increased significantly in the Δ11-1 Δ11-4 and Δ11-5 mutants (~44, ~27 and ~22% each). In addition, the Δ11-3 and Δ11-6 mutants showed decreased exon 11 inclusion (~33 and ~33% each). Thus, we concluded that the RNA length (20 nt) and the sequences of 11-3 and 11-6 contained an enhancer for exon 11 inclusion, whereas 11-1, 11-4 and 11-5 RNA contained an inhibitor for exon 11 inclusion of Ron pre-mRNA. Since the Δ11-6 mutant produced a product caused by partial splicing (shown as *), the 11-6 RNA part probably also regulated the partial splicing. To further identify the enhancer for exon 11 inclusion, we selected the 11-3 RNA part for further study.

### The 2nd 10 nt but not the 1st 10 nt in 11-3 functions as an enhancer for exon 11 inclusion

**Figure 2:**
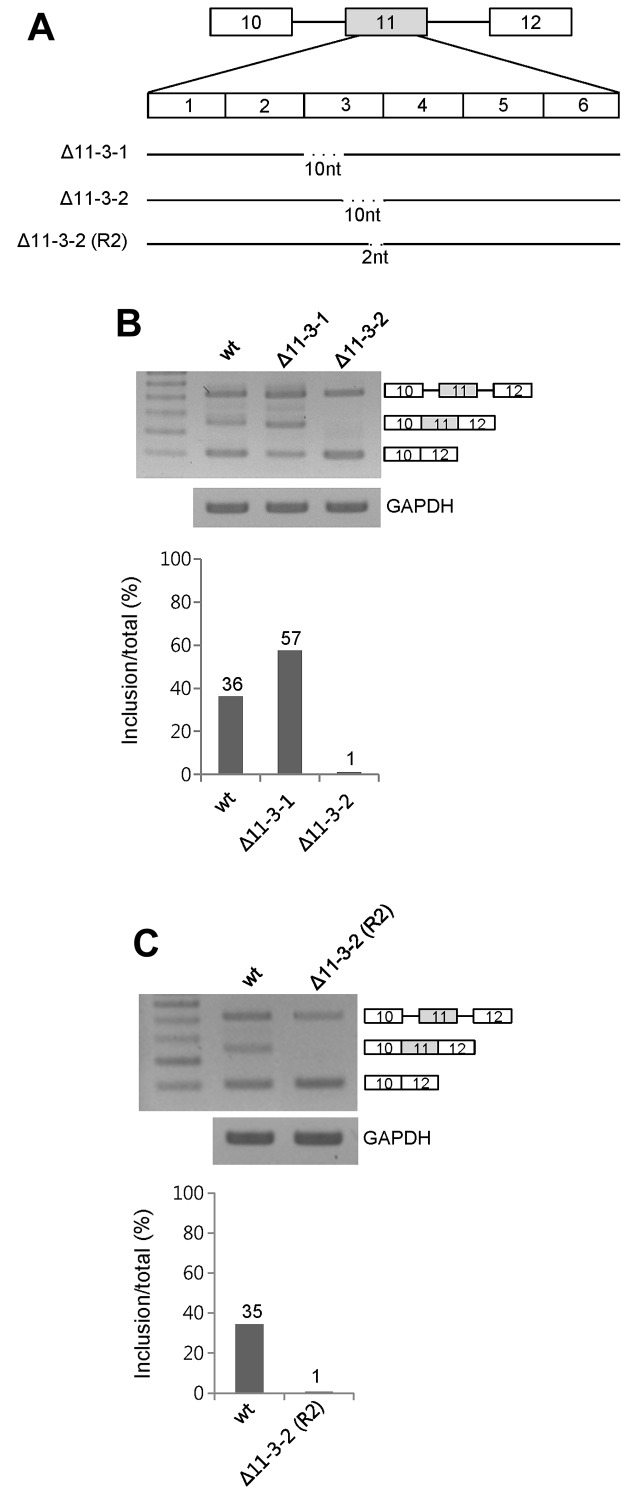
The 2nd 10 nt but not the 1st 10 nt in 11-3 functions as an enhancer for exon 11 inclusion. (A) Deletion mutants (Δ11-3-1 and Δ11-3-2) and [Δ11-3-2(R2)] are shown. The 10-nt and 2-nt RNAs were deleted in each mutant. (B and C) RT-PCR analysis of wild-type (wt) minigene and deletion mutants. Quantitation of the results is shown as the ratio of exon 11 inclusion to total RNA.

To further identify the enhancer for exon 11 inclusion in 11-3 RNA of exon 11, we dissected 20 nt of 11-3 RNA into two 10-nt RNA sections. The two 10-nt deleted mutants were produced as shown in Fig. 2A, labeled Δ11-3-1 and Δ11-3-2. RT-PCR analysis showed that only the Δ11-3-2 mutant decreased exon 11 inclusion (~35%) whereas Δ11-3-1 increased exon 11 inclusion (~21%) (Fig. 2B). Therefore, we conclude that 10 nt of the 11-3-2 RNA includes the enhancer for exon 11 inclusion.

### The 2-nt RNA at the 3′ end of the 11-3-2 RNA functions as an enhancer for exon 11 inclusion

**Figure 3:**
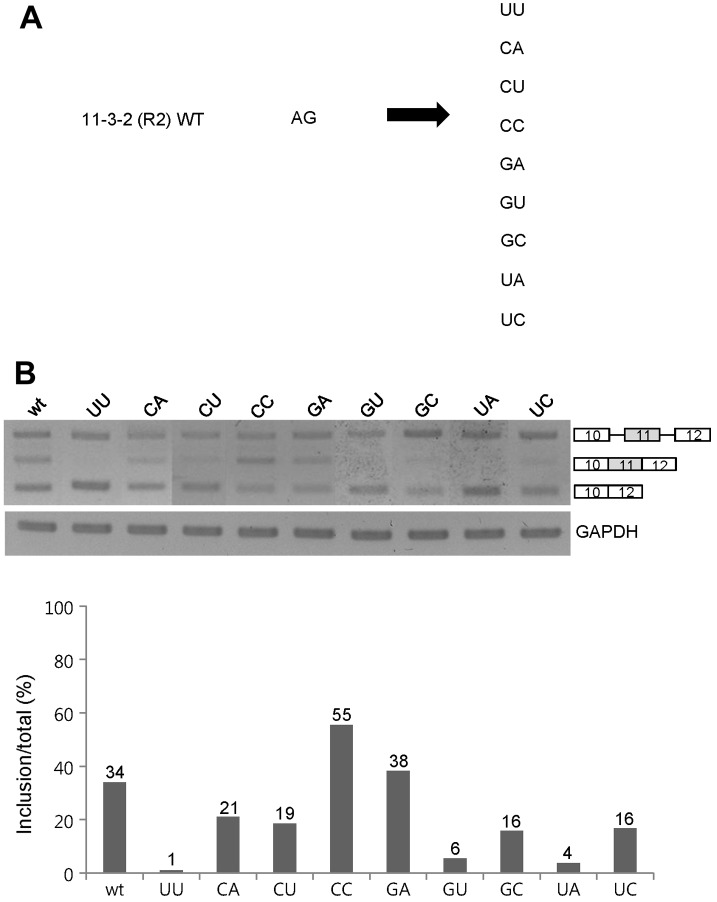
GA, CC and the wild-type AG sequences function as enhancers for exon 11 inclusion of Ron pre-mRNA. (A) Both nucleotides of AG [11-3-2 (R2)] were mutated to other sequences. The mutated RNA sequences in each mutant are shown. (B) RT-PCR analysis of wild-type (wt) minigene and mutants. Quantitation of the results of RT-PCR is shown.

To further understand the enhancer for exon 11 inclusion, we dissected the 2nd 10-nt RNA of 11-3. We deleted 2 nt from the 3′ end of the 10-nt RNA, labeled Δ11-3-2 (R2) (Fig. 2A). After extraction of RNA from the minigene-transfected cells, we performed RT-PCR. The results in Fig. 3B demonstrated that the Δ11-3-2 (R2) mutant expressed the exon 11 skipped form exclusively (Fig. 2C). Therefore, we concluded that the 2-nt RNA at the 3′ end of the 11-3-2 RNA contains enhancers for exon 11 inclusion.

### GA, CC and the wild-type AG sequences function as enhancers for exon 11 inclusion of Ron pre-mRNA

Since the 2-nt RNA at the 11-3-2 section on exon 11 acts as a strong enhancer for exon 11 inclusion, we decided to pinpoint the sequence requirements for the 2-nt RNA. As the first approach, we performed substitution mutagenesis analysis on both nucleotides of the 2-nt RNA. We mutated the AG sequence into various sequences that cover all of the four base combinations (Fig. 3A). Among the mutants, as shown in Fig. 3B, exon 11 inclusion was completely compromised in the UU, GU and UA mutants. The CA, CU, GC and UC mutants showed a significant decrease in exon 11 inclusions (~13, ~15, ~18 and ~18%). However, exon 11 inclusion was increased in the CC mutant, whereas the GA mutant showed a comparable level of exon 11 inclusion as the wild-type minigene. The substitution mutant results indicate that most 2-nt sequences promoted exon 11 skipping of Ron pre-mRNA. One opposite case was the CC RNA sequence that promoted exon 11 inclusion (~21%). Therefore, we concluded that the GA, CC and AG sequences at the 3′ end of 11-3-2 RNA are required for the function of the 2-nt RNA as an enhancer for exon 11 inclusion of Ron pre-mRNA.

### UG and AC also function as enhancers for exon 11 inclusion of Ron pre-mRNA

**Figure 4:**
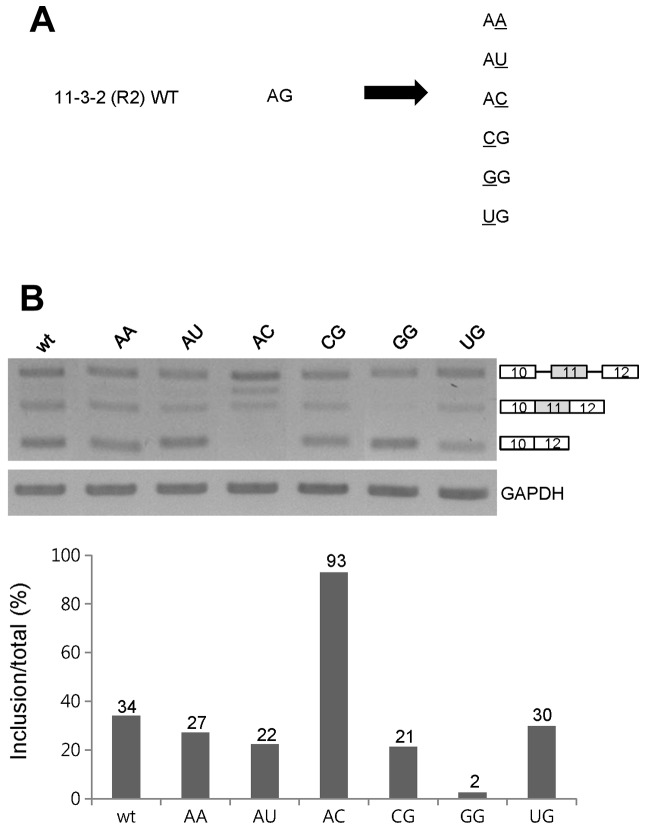
UG and AC also function as enhancers for exon 11 inclusion of Ron pre-mRNA. (A) The 1 nt of AG was mutated in each mutant. The mutated RNA sequences in each mutant are shown. (B) RT-PCR analysis of wild-type (wt)minigene and mutants. Quantitation of the results of RT-PCR is shown.

In order to further understand the sequence requirement of the 2-nt enhancer at the 3′ end of the 11-3-2 RNA, as the second approach, we performed single nucleotide substitution mutagenesis analysis. In the first set of single nucleotide mutagenesis assay, we mutated the A nucleotide of wild-type AG at the 11-3-2 (R2) RNA into the C, G or U nucleotide, whereas the G residue at the wild-type AG remain unchanged, and were named as CG, 1GG and UG. In the second set of mutagenesis, we mutated the G nucleotide of the AG dinucleotide into A, U and C nucleotides separately, whereas the A residue of AG remained unchanged, and were named as AA, AU and AC (Fig. 4A). RT-PCR analysis in Fig. 4B shows that the GG mutant demonstrated a completely compromised exon 11 inclusion. In addition, exon 11 inclusion was significantly reduced in the CG and UG mutant (~13%). In contrast, exon 11 skipping was not significantly decreased in the UG mutant (~3%). Thus, to maintain the enhancer function of the wild-type AG dinucleotide for exon 11 inclusion, the first position should be A and U but not C and G residue. In the second residue substitution mutants, as shown in Fig. 4B, the AC mutant, in which the G nucleotide of the AG dinucleotide was substituted by the C residue, showed the exon 11 inclusion form exclusively (~93%). Thus, the AC sequence functions as a stronger enhancer. However, exon 11 inclusion was reduced in the AU and AA mutants (~12 and ~7%). Therefore, we conclude that the G or C nucleotide at the second position of the AG dinucleotide maintains or increases its enhancer function for exon 11 inclusion. Collectively, the A or U residue at the first position, in combination with G or C at the second position of the wild-type AG dinucleotide are required for the enhancer function for exon 11 inclusion. We summarize that UG and AC function as enhancers for exon 11 inclusion of Ron pre-mRNA.

## Discussion

Ron proto-oncogene, a receptor tyrosine kinase, produces the Δ165 isoform through exon 11 skipping. The Δ165 isoform is a constitutively active isoform without the binding of the MSP ligand. Exon 11 inclusion is regulated by the juxtaposed enhancers and inhibitors which are located at exon 12. We identified a 2-nt enhancer for exon 11 inclusion at exon 11, located at 74 nt upstream from the 5′ splice site of exon 11, through serial deletion analysis. Through double base substitution analysis, we demonstrated that, in addition to the AG sequence, GA and CC also maintained their enhancer function. Furthermore, through the single base substitution analysis, we found that UG and AC function as enhancers for exon 11 inclusion of Ron pre-mRNA.

### Exon 11 inclusion/skipping is regulated by multiple cis-acting elements

Previously, it was shown that an enhancer is located at exon 12 to promote exon 11 skipping. It was also shown that the inhibitor RNA, which is located next to the enhancer, promotes exon 11 inclusion. Most importantly, the antagonistic effects of the enhancer and inhibitor regulate exon 11 inclusion and skipping. Our results here demonstrated that exon 11 inclusion/skipping is regulated by, in addition to the enhancer and inhibitor on exon 12, the enhancer on exon 11. Through multiple 20-nt deletion analyses, we found that different 20-nt deletions had different effects on exon 11 splicing.

Exon 11 inclusion was increased significantly in the Δ11-1, Δ11-4 and Δ11-5 mutants, was decreased significantly in the Δ11-3 and Δ11-6 mutants, and remained at a similar level for the wild-type minigene in the Δ11-2 mutant. Our results indicate that most deletion mutations of exon 11 showed the alteration of exon 11 inclusion. Thus, we concluded that exon inclusion/skipping is regulated by multiple *cis*-elements.

### Simple determination of the exon enhancer by deletion mutagenesis is not always correct

Our deletion mutagenesis analysis showed that the 20-nt RNA (11-3) had the enhancer function (Fig. 1B). However, through further deletion we found that the upstream 10 nt had an inhibitor function, whereas the downstream 10 nt functioned as an enhancer (Fig. 2B). Thus, it is not correct to determine the splicing enhancer by deletion mutagenesis, although deletion mutagenesis definitely provides important information. *cis*-acting elements of pre-mRNA splicing are composed of different combinations of four nucleotides, and usually provide the functional targets for *trans*-acting elements. It is not surprising that each 10-nt RNA had the opposite functions on exon 11 inclusion. One possibility is that one 10-nt RNA section provided the contact for activator proteins, and the other one provided the contact for the inhibitory proteins. Another possibility is that the deletion of 10 nt made the flanking sequences to be connected to produce another enhancer sequence. Therefore, determination of splicing enhancer by deletion mutagenesis is not always correct.

### Length and sequence of RNA play roles in exon 11 inclusion/skipping

Our substitution analysis of the AG dinucleotides demonstrated that different bases had different effects on exon 11 inclusion of Ron pre-mRNA. By double nucleotide mutagenesis, we found that the GA, CC as well as AG sequences at the 3′ end of the 11-3-2 RNA were required for the function of the 2-nt RNA as an enhancer for exon 11 inclusion of Ron pre-mRNA. By single base substitution analysis, we found that the A or U residue at the first position, and the G or C at the second position of the AG dinucleotide were required for the enhancer function for exon 11 inclusion. Surprisingly, we found that several mutants (UA, GC, UU and GG) completely destroyed exon 11 inclusion, whereas the AC mutant completely destroyed exon 11 skipping. Our results indicate the high sequence requirement of the enhancer for exon 11 inclusion of Ron pre-mRNA.

## Figures and Tables

**Table I:** The primers used.

Name	Sequences (5′-3′)
RON10-*Hin*dIII-for	ATGTTAAGCTTCCTGAATATGTGGTCCGAGAC
RON12-*Xho*I-rev	CTTACCTCGAGCTAGCTGCTTCCTCCGCCACC
Δ11-1-for	TATATTGGGCTGGGCTATCAACGTGACCGT
Δ11-1-rev	ACGGTCACGTTGATAGCCCAGCCCAATATA
Δ11-2-for	GGCTGACTGTGTGGGGTGAGAGCTGCCAGC
Δ11-2-rev	GCTGGCAGCTCTCACCCCACACAGTCAGCC
Δ11-3-for	ACGTGACCGTGGGTGTTCCGGGGGGACATG
Δ11-3-rev	CATGTCCCCCCGGAACACCCACGGTCACGT
Δ11-4-for	AGCTGCCAGCACGAGCTGCCCCCTGCCCCC
Δ11-4-rev	GGGGGCAGGGGGCAGCTCGTGCTGGCAGCT
Δ11-5-for	GGGGGACATGGTTGTTGCAGCTTGGCCAGG
Δ11-5-rev	CCTGGCCAAGCTGCAACAACCATGTCCCCC
Δ11-6-for	CCCTGCCCCCATCCCGGTGCCCCATTGCAG
Δ11-6-rev	CTGCAATGGGGCACCGGGATGGGGGCAGGG
Δ11-3-1-for	ACGTGACCGTGGGTGCCAGCACGAGTTCCG
Δ11-3-1-rev	CGGAACTCGTGCTGGCACCCACGGTCACGT
Δ11-3-2-for	GGGTGGTGAGAGCTGTTCCGGGGGGACATG
Δ11-3-2-rev	CATGTCCCCCCGGAACAGCTCTCACCACCC
Δ11-3-2(R2)-for	CTGCCAGCACGTTCCGGGGGGA
Δ11-3-2(R2)-rev	TCCCCCCGGAACGTGCTGGCAG
UU-for	CTGCCAGCACGTTTTCCGGGGGGA
UU-rev	TCCCCCCGGAAAACGTGCTGGCAG
CA-for	CTGCCAGCACGCATTCCGGGGGGA
CA-rev	TCCCCCCGGAATGCGTGCTGGCAG
CU-for	CTGCCAGCACGCTTTCCGGGGGGA
CU-rev	TCCCCCCGGAAAGCGTGCTGGCAG
CC-for	CTGCCAGCACGCCTTCCGGGGGGA
CC-rev	TCCCCCCGGAAGGCGTGCTGGCAG
GA-for	CTGCCAGCACGGATTCCGGGGGGA
GA-rev	TCCCCCCGGAATCCGTGCTGGCAG
GU-for	CTGCCAGCACGGTTTCCGGGGGGA
GU-rev	TCCCCCCGGAAACCGTGCTGGCAG
GC-for	CTGCCAGCACGGCTTCCGGGGGGA
GC-rev	TCCCCCCGGAAGCCGTGCTGGCAG
UA-for	CTGCCAGCACGTATTCCGGGGGGA
UA-rev	TCCCCCCGGAATACGTGCTGGCAG
UC-for	CTGCCAGCACGTCTTCCGGGGGGA
UC-rev	TCCCCCCGGAAGACGTGCTGGCAG
AA-for	CTGCCAGCACGAATTCCGGGGGGA
AA-rev	TCCCCCCGGAATTCGTGCTGGCAG
AU-for	CTGCCAGCACGATTTCCGGGGGGA
AU-rev	TCCCCCCGGAAATCGTGCTGGCAG
AC-for	CTGCCAGCACGACTTCCGGGGGGA
AC-rev	TCCCCCCGGAAGTCGTGCTGGCAG
CG-for	CTGCCAGCACGCGTTCCGGGGGGA
CG-rev	TCCCCCCGGAACGCGTGCTGGCAG
GG-for	CTGCCAGCACGGGTTCCGGGGGGA
GG-rev	TCCCCCCGGAACCCGTGCTGGCAG
UG-for	CTGCCAGCACGTGTTCCGGGGGGA
UG-rev	TCCCCCCGGAACACGTGCTGGCAG
